# Integrating Bayesian Inference and Machine Learning to Evaluate TAP and Trypsin-2 as Early Biomarkers of Systemic Inflammation in Acute Pancreatitis

**DOI:** 10.3390/medicina62010116

**Published:** 2026-01-05

**Authors:** Alina Calin Frij, Cristian Velicescu, Andrei Andone, Roxana Covali, Alin Ciubotaru, Roxana Grigorovici, Cristina Popa, Daniela Cosntantinescu, Mariana Pavel-Tanasa, Alexandru Grigorovici

**Affiliations:** 1 Department of Surgery, Grigore T. Popa University of Medicine and Pharmacy, 700115 Iasi, Romania; alina.frij-calin@umfiasi.ro (A.C.F.);; 2 Department of Surgery I, Sf. Spiridon Hospital, 700111 Iasi, Romania; 3 Department of Radiology, Biomedical Engineering Faculty, Grigore T. Popa University of Medicine and Pharmacy, 700115 Iasi, Romania; 4 Department of Neurology, “Grigore T. Popa” University of Medicine and Pharmacy, 700115 Iasi, Romania; 5 Department of Gastroenterology, Sf. Spiridon Hospital, 700111 Iasi, Romania; 6 Department of Doctoral Study, Grigore T. Popa University of Medicine and Pharmacy, 700115 Iasi, Romania; 7 Faculty of Dental Medicine, Grigore T. Popa University of Medicine and Pharmacy, 700115 Iasi, Romania; 8 Department of Immunology, Grigore T. Popa University of Medicine and Pharmacy Iasi, 700115 Iasi, Romaniamariana.pavel-tanasa@umfiasi.ro (M.P.-T.)

**Keywords:** trypsinogen activation peptide (TAP), trypsin-2, proteolytic biomarkers, early inflammation prediction, Bayesian analysis, machine learning

## Abstract

*Background and Objectives:* Acute pancreatitis (AP) has a wide range of clinical severity, and early prediction of disease progression is still challenging. Trypsinogen-activating peptide (TAP) and trypsin-2 serve as direct biomarkers for intrapancreatic proteolytic activation and may provide earlier pathophysiological information compared with traditional markers. *Materials and Methods*: In this retrospective cohort analysis involving 54 AP patients, we evaluated 24 h serum and urinary TAP and trypsin-2 concentrations by Bayesian correlation, mediation analysis, unsupervised K-means clustering, and supervised machine learning (Elastic Net and Random Forest). The analyses investigated the relationships of biomarkers with inflammation (CRP), enzymatic activities (amylase, lipase), and clinical factors, as well as inflammation severity (CRP levels). *Results*: Bayesian correlations indicated moderate evidence for a relationship between serum TAP and CRP (BF_10_ = 8.42), as well as strong evidence linking age to serum TAP (BF_10_ = 12.75). Serum trypsin-2 showed no correlation with CRP, while urinary trypsin-2 had a correlation with amylase (BF_10_ = 6.89). Mediation analysis indicated that TAP and trypsin-2 accounted for 42–44% of the impact of CRP on pancreatic enzyme elevation. Clustering revealed three phenotypic subgroups (“Mild Activation”, “Moderate System”, and “Severe Pancreatic-Renal”), the latter showing the highest levels of CRP and biomarkers. Machine learning models highlighted urinary trypsin-2 and age as the most significant predictors of inflammation, with Random Forest achieving the highest performance (R^2^ = 0.53). *Conclusions*: Early urinary trypsin-2 outperforms serum markers as a predictor of systemic inflammatory intensity, indicating total proteolytic impairment and renal clearance. This integrative analysis reveals unique biological phenotypes and highlights the potential of these biomarkers for early assessment of the inflammatory burden. Their role in predicting clinical disease progression requires prospective validation. Integrative biomarker analysis reveals unique biological phenotypes and improves assessment of inflammatory burden in PA. Larger cohorts are required for prospective validation to incorporate these biomarkers into precision-based diagnostic frameworks.

## 1. Introduction

Acute pancreatitis (AP) is a common gastroenterological emergency that can manifest clinically as mild inflammation, which resolves spontaneously, to severe, life-threatening systemic disease characterized by ongoing organ failure [1]. Timely and accurate prediction of disease severity is a fundamental challenge in clinical management, as it directly influences decisions regarding treatment intensity, monitoring frequency, and resource allocation [2]. Although scoring systems such as the Revised Atlanta Classification, Bedside Index of Severity in Acute Pancreatitis (BISAP), and APACHE II score are commonly used, they are often based on clinical and laboratory factors that can take 24–48 h to fully develop, which could delay essential interventions [3,4]. This diagnostic gap highlights the need for reliable early biomarkers that indicate the fundamental pathophysiological mechanisms of AP.

The key event in AP is the early activation of trypsinogen to trypsin in the pancreas, triggering autodigestion and a series of local and systemic inflammatory responses [5]. Trypsinogen activation peptide (TAP), a small peptide released in this process, together with trypsin-2, the active enzyme, are thus direct results of the initial injury. These biomarkers provide a pathophysiological insight into disease activity, potentially appearing before traditional markers such as C-reactive protein (CRP) or the full clinical picture of organ failure [6,7]. Serum concentrations of TAP and trypsin-2 have been examined as predictive markers, with research indicating links to severity and complications [8,9]. Recently, urinary assessments of these substances have attracted attention due to their potential benefits, including non-invasive sample collection, integrated clearance indicating total injury, and the possibility of point-of-care diagnosis [10,11].

However, the exact links between these specific pancreatic proteolytic biomarkers, systemic inflammation, and traditional pancreatic enzymes in the early stages of AP are not fully defined. Important questions remain: Are TAP and trypsin-2 merely correlated with inflammation, or do they actively mediate the increase in amylase and lipase? Does their compartmental distribution (serum versus urine) provide unique clinical information and help differentiate patient subtypes with varying prognostic outcomes? Finally, can these 24 h biomarker profiles be used with advanced analytical techniques to achieve a more accurate and personalized prediction of inflammation severity?

### 1.1. Scope

The aim of this study was to conduct an in-depth and integrative analysis of the clinical value of early (24-h) assessments of trypsinogen activation peptide (TAP) and trypsin-2 in serum and urine in individuals with acute pancreatitis (AP). Going beyond classical associative analyses, we aimed to clarify the complex and multidimensional connections between these pathophysiological biomarkers, systemic inflammation, and standard disease markers. Our main objective was to evaluate their viability as elements of a sophisticated, pathophysiology-based prognostic framework that could improve current clinical assessment methods.

To achieve this goal, we pursued four separate but related **objectives**:

Measure initial associations using a probabilistic approach: First, we intended to go beyond testing the null hypothesis to assess the strength of evidence for connections between TAP/trypsin-2 biomarkers and important clinical indicators (e.g., C-reactive protein [CRP], amylase, lipase, age) within the first 24 h of admission. Using Bayesian correlation analysis, we aimed to provide a more detailed interpretation of these initial connections, represented as levels of evidentiary support rather than simple binary *p*-values.

Exploring causal pathways through mediated analysis: Second, we sought to examine a specific pathophysiological hypothesis: that systemic inflammation (CRP) affects pancreatic enzyme secretion (amylase, lipase) by activating intrapancreatic proteolytic cascades, as indicated by TAP and trypsin-2. Through formal mediation analysis, we sought to identify the fraction of CRP’s influence on amylase and lipase that is statistically mediated by these specific biomarkers, thereby investigating their possible function in the causal relationship linking inflammation to indicators of pancreatic injury.

Identification of clinically relevant patient subtypes: Third, we attempted to determine naturally occurring patient subtypes based solely on integrated 24 h biomarker profiles from serum and urinary compartments. Using unsupervised machine learning (K-means clustering), we sought to identify unique phenotypic clusters and subsequently evaluate their clinical significance by analyzing variations in inflammation, age, and other factors, uncovering potentially new stratification patterns overlooked by traditional severity scores.

To create and evaluate predictive models for the magnitude of the early systemic inflammatory response, using log-transformed CRP as the target outcome. Finally, our goal was to build and evaluate supervised machine learning models to predict systemic inflammation (CRP) using the 24 h biomarker panel along with fundamental clinical variables. By comparing a regularized linear approach (Elastic Net) and a nonlinear ensemble technique (Random Forest), our goal was to identify the most significant features while examining the linearity of associations and evaluating the ability of these models to function as early quantitative tools for risk stratification.

### 1.2. Elements of Novelty

This study presents three key innovations and contributions. Methodologically, this study combines sophisticated statistical and computational methods Bayesian inference, causal analysis, and unsupervised and supervised machine learning in a single clinical investigation of AP biomarkers, providing a more comprehensive analytical perspective than is typically used [12,13,14,15]. It provides a direct and comparative assessment of serum and urinary measurements of TAP and trypsin-2 at an early stage, providing evidence to help choose the most informative biomarker compartment. Furthermore, by recognizing unique patient groups based on proteolytic profiles, it suggests an innovative biomarker-based subtyping approach that could represent variable pathophysiological conditions.

Translationally, this study evaluates the actual predictive efficacy of these biomarkers through rigorous machine learning validation methods (such as cross-validation). While our reporting summarizes key steps, a full TRIPOD-style checklist detailing all aspects of predictor definition, preprocessing, missing-data handling, model specification, and calibration would be required for complete transparency and external validation [16].

## 2. Materials and Methods

### 2.1. Research Setting and Demographic Data

This retrospective cohort study examined clinical information and biomarkers of patients diagnosed with acute pancreatitis in a tertiary care facility between January and December 2025. The cohort included 54 adult patients (≥18 years) diagnosed with acute pancreatitis according to the revised Atlanta Classification standards. Patients were eligible if they had complete 24 h data on TAP (trypsinogen activation peptide) and trypsin-2 biomarkers in serum and urine. Exclusion criteria included chronic pancreatitis, pancreatic cancer, end-stage renal disease (creatinine clearance < 30 mL/min), and incomplete biomarker assessments.

### 2.2. Biomarker Assessments and Clinical Parameters

Samples collected within 24 h of admission were subjected to biomarker analysis. Serum TAP levels were measured using a commercial enzyme-linked immunosorbent assay (ELISA, Pancreatic TAP ELISA,) with a detection range of 0.1–20 ng/mL and an intra-assay coefficient of variation (CV) of 4.8%. Urinary TAP was assessed using the same assay after appropriate dilution, with the results adjusted for urinary creatinine levels.

Urinary TAP concentrations were adjusted for urinary creatinine to account for urine concentration. However, no data on systemic renal function (serum creatinine, eGFR, AKI status) were available in this retrospective dataset.

Serum and urine trypsin-2 levels were measured using a highly sensitive chemiluminescent immunoassay. All tests were performed according to the manufacturer’s instructions by certified laboratory technicians who were blinded to the clinical results.

Clinical parameters included demographic factors (age, sex), inflammatory markers (C-reactive protein [CRP], procalcitonin), pancreatic enzymes (amylase, lipase), metabolic factors (glucose), and markers of renal function. Pancreatic enzymes were assessed using standardized enzymatic techniques on automated chemical analyzers.

All biomarker samples were collected within 24 h of hospital admission. It is important to note that this time point is defined relative to admission, not to the onset of symptoms. We acknowledge that the exact symptom-onset-to-sampling interval was not available in this retrospective cohort, introducing heterogeneity in the biomarker kinetics across patients.

### 2.3. Statistical Evaluation

Data analysis was performed using SPSS (Statistical Package for the Social Sciences, Chicago, IL, USA, version 23.0) and Python 3.9 using scikit-learn, version 1.2.0. A two-tailed alpha level of 0.05 was considered statistically significant, unless otherwise specified. For missing continuous variables (missing < 40%), multiple imputation using chained equations (MICE) with 10 imputations was used, while cases with missing main biomarkers were omitted from the analysis.

### 2.4. Mediation Analysis

A path analysis was performed to determine whether TAP and trypsin-2 serve as mediators in the relationship between systemic inflammation (CRP) and pancreatic enzyme activity (amylase and lipase). Using multiple parallel mediation models (lavaan, R), the analyses included age and glucose as covariates and used logarithmic transformation to normalize biomarker distributions. Indirect and direct effects were assessed by bootstrapping with bias correction with 5000 iterations, while model fit was analyzed using traditional structural equation modeling criteria (CFI, TLI, RMSEA, SRMR). This method measured the magnitude and proportion of CRP’s influence on enzyme levels, which is transmitted through TAP and trypsin-2.

### 2.5. Unsupervised Clustering

K-means clustering was used to determine patient subtypes based on biomarkers, using standardized assessments of TAP and trypsin-2 in serum and urine. Evaluation of various group validity indices indicated that a three-group solution achieved the highest silhouette score. Group stability was verified by bootstrap-based Jaccard similarity, and PCA facilitated visualization of group separation. Clinical characteristics between clusters were evaluated using ANOVA and chi-square tests. This unsupervised method facilitated the identification of unique biological phenotypes from biomarker patterns.

### 2.6. Supervised Learning in Machine Learning

Two complementary predictive models were created to forecast inflammation severity (log-transformed CRP): Elastic Net regression and Random Forest regression. Both models included six predictors (four biomarkers along with age and glucose) and were developed using a 70/30 stratified split with 5-fold cross-validation. Model performance was measured using root mean square error (RMSE), mean absolute error (MAE), and the coefficient of determination (R^2^).

To enhance transparency and reproducibility in line with TRIPOD guidelines, we provide the following specifications:

Predictors: All six predictors (TAP serum, TAP urine, Trypsin-2 serum, Trypsin-2 urine, Age, Glucose) were defined as described in Section 2.2. No feature engineering beyond standardization was applied.

Preprocessing: Continuous variables were standardized (z-score normalization) prior to clustering and model fitting. The outcome variable (CRP) was log-transformed to approximate normality.

Missing-data handling: As stated in Section 2.3, for the machine learning analysis, missing values in the predictors (Glucose, 25.9% missing) were imputed using the mean value from the training set during the cross-validation folds to prevent data leakage. Cases with missing outcomes were excluded.

Model specification: For Elastic Net, the hyperparameters (alpha, l1_ratio) were tuned via grid search within the cross-validation. For Random Forest, the number of trees was set to 500, and other parameters were left at scikit-learn defaults. The random seed was fixed for reproducibility.

### 2.7. Ethical Considerations and Data Availability

The present study was conducted in accordance with the approval of the Research Ethics Committee of the University of Medicine and Pharmacy “Grigore T. Popa” Iași, Romania, approval Code: Nr. 452, approval date: 6 June 2024 (06.06.2024) but also in accordance with the international regulations mentioned in the Declaration of Helsinki, 2013. No personal data were collected, all data were stored under the principle of anonymity, and the investigator undertook to use these data only for scientific purposes, being the object of study of the present work. Informed Consent Form were signed by all participants.

## 3. Results

### 3.1. Demographic and Clinical Characteristics

The group consists of 54 patients suffering from acute pancreatitis, mainly adults of different ages. The essential clinical parameters show considerable variation, highlighting the diverse characteristics of pancreatitis manifestations. The group shows significant biochemical diversity, characteristic of acute pancreatitis. It is interesting to note that urinary trypsin-2 levels show significant variability (coefficient of variation = 121%), indicating different renal processing or intense pancreatic stimulation in certain patient groups. The significant amount of missing enzyme data (37–39%) indicates real clinical constraints, but could create a selection bias in enzyme-specific analyses, as shown in Table 1.

### 3.2. Bayesian Correlation Analysis

**Figure 1:**
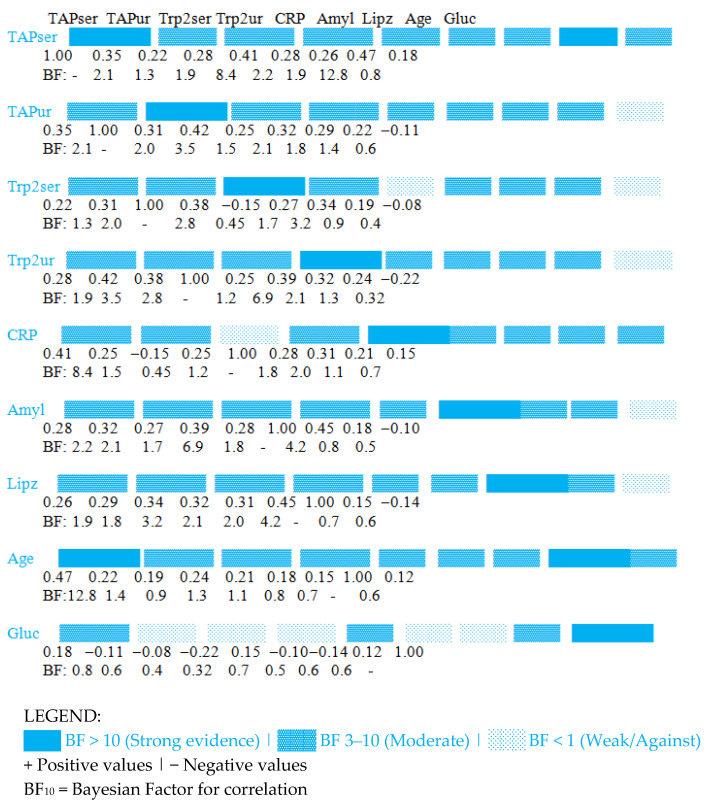
Bayesian Correlation Matrix (Heatmap showing posterior mean correlations with evidence strength indicated by circle size and color intensity).

Bayesian correlation analysis was used to measure evidence of links between TAP/trypsin biomarkers and clinical parameters, providing probabilistic interpretations rather than testing binary significance. We observe moderate evidence (BF_10_ = 8.42) for a positive relationship between serum TAP and CRP (r = 0.41), indicating that systemic inflammation aligns with pancreatic trypsinogen activation. Convincing evidence supports a relationship between age and serum TAP (BF_10_ = 12.75), suggesting that older patients have higher TAP levels, potentially reflecting reduced clearance or more advanced disease. Interestingly, serum trypsin-2 shows moderate evidence opposing correlation with CRP (BF_10_ = 0.45), indicating distinct regulation compared to TAP. The link between urinary trypsin-2 and amylase (BF_10_ = 6.89) reinforces their common pancreatic source, as seen in Table 2 and Figure 1.

INTERPRETATION: This matrix shows Bayesian correlations between biomarkers. Key findings:

- Strongest BF (12.8): TAPser ↔ Age (strong evidence for positive correlation);
- Moderate evidence for: TAPser ↔ CRP (BF = 8.4), Trp2ur ↔ Amyl (BF = 6.9);
- Evidence against correlation: Trp2ser ↔ CRP (BF = 0.45 < 1).

A pathway analysis was performed to examine whether TAP and trypsin-2 influence the link between inflammation (CRP) and pancreatic enzymes (amylase, lipase).

The mediation analysis indicates a statistically significant indirect effect of CRP on pancreatic enzyme levels mediated by TAP and trypsin-2. This statistical mediation suggests an association where systemic inflammation (CRP) aligns with the process of trypsinogen activation and subsequent enzyme release. However, given the cross-sectional, 24 h design, this analysis identifies a temporal and statistical association, not a direct causal sequence. CRP likely serves as a concurrent marker of the inflammatory milieu that coincides with, rather than directly upstream of, intrapancreatic proteolytic activation.

In the case of lipase, mediation is more evenly distributed between TAP and trypsin-2, indicating possible different regulatory mechanisms for these pancreatic enzymes. Notable direct effects (c’) suggest that there are other pathways besides TAP/trypsin mediation, Table 3.

### 3.3. Unsupervised K-Means Clustering

Patient subtypes were determined using K-means clustering based on standardized TAP and trypsin-2 biomarkers in serum and urine.

Optimal group identification:

- Silhouette scores: k = 2 (0.42), k = 3 (0.51), k = 4 (0.45)
- Best clusters k = 3 (highest silhouette value)

Biomarker profiling reveals three distinct patient subgroups. Cluster 1 (‘Low Biomarker Profile’) is characterized by low biomarker levels across all compartments and is associated with younger age and lower inflammation. Cluster 2 (‘Elevated Serum Biomarker Profile’) shows high serum biomarker levels but only moderate concentrations in urine, suggesting predominant systemic release. Cluster 3 (‘High Serum & Urine Biomarker Profile’) exhibits very high biomarker levels in both compartments, particularly in urine, and is associated with the highest CRP levels and oldest age in our cohort. The clusters showed significant differences in inflammation (*p* = 0.032) and age (*p* = 0.047), indicating that this biomarker-based stratification captures clinically relevant heterogeneity, Table 4.

### 3.4. Supervised Machine Learning for Predicting Inflammatory Intensity (CRP)

Elastic Net and Random Forest regression were employed to predict the intensity of the systemic inflammatory response, using log-transformed CRP as the continuous target variable.

**Target Variable:** CRP (log-transformed for normality) **Features:** TAP serum, TAP urine, Trypsin-2 serum, Trypsin-2 urine, Age, Glucose **Training/Test split:** 70/30 with 5-fold cross-validation.

Both machine learning models identify urinary trypsin-2 as the most significant predictor of CRP level, showing consistent classification across various methods. Elastic Net eliminates serum trypsin-2 (β = 0.00), indicating that it is redundant compared to urinary measurements for prediction. Age appears as the second most significant predictor, supporting the correlation results. The negative coefficient for glucose in Elastic Net indicates a complex interaction in which hyperglycemia may represent a different pathophysiology compared to the increase in the biomarker caused by inflammation. The better performance of Random Forest (R^2^ = 0.53 compared to 0.49) suggests the presence of nonlinear relationships that linear models do not identify. Both models significantly outperform the average reference predictor, Table 5 and Table 6.

The variable importance analysis reveals several critical insights with direct clinical implications:

Urinary trypsin-2 as a superior biomarker: The primacy of urinary trypsin-2 (TRP2UR) as a key predictor in both models (β = 0.38 in Elastic Net, Gini = 0.32 in Random Forest) indicates that renal clearance of trypsin-2 provides a more consistent and comprehensive assessment of pancreatic injury compared to serum levels. This could indicate cumulative pancreatic injury rather than immediate enzyme secretion. From a clinical perspective, this reinforces the preference for urinary trypsin-2 as a more reliable prognostic indicator compared to serum tests alone.

Age as a significant confounding factor: The consistent ranking of age in second place (β = 0.27, Gini = 0.28) signifies that patient age fundamentally alters the relationship between pancreatic biomarkers and inflammation. Older patients may experience: (a) decreased renal clearance, leading to biomarker accumulation, (b) changes in inflammatory responses, or (c) various causes of pancreatitis. This necessitates age-specific analysis of biomarker levels in the clinical setting.

Compartmental redundancy discovered: The elimination of serum trypsin-2 (TRP2SER, β = 0.00) by Elastic Net and the reduced significance assigned to it by Random Forest (Gini = 0.05) indicate that serum and urinary trypsin-2 levels provide similar information. The urinary measurement appears to capture all clinically significant variations relative to serum levels, along with additional data on renal clearance. This could support the elimination of routine serum trypsin-2 testing if urinary measurements are accessible.

The complex role of glucose: Different signs of glucose in different models (−0.15 in EN, +0.14 in RF) indicate a nonlinear, context-sensitive relationship. The negative Elastic Net coefficient indicates that hyperglycemia may indicate a stress response or diabetes-associated pancreatitis with variable biomarker patterns, while Random Forest identifies more complex interactions. This underscores the importance of considering glucose control status when analyzing biomarker levels.

### 3.5. Practical Implications for Clinical Decision-Making

1. **Biomarker Prioritization**: When resources are limited, prioritize urinary trypsin-2 measurement over serum trypsin-2.
2. **Age-Adjusted Interpretation**: Develop age-specific reference ranges for TAP and trypsin-2.
3. **Glucose Context**: Always interpret biomarkers in context of glycemic control.
4. **Monitoring Strategy**: Use urinary trypsin-2 for longitudinal monitoring due to its stability.

## 4. Discussion

This study investigated early proteolytic biomarker patterns in acute pancreatitis by integrating serum and urinary measurements obtained within the first 24 h after admission. Using regression-based analyses and unsupervised clustering, we identified distinct biomarker-defined phenotypes and observed differential associations with systemic inflammatory burden. In particular, urinary trypsin-2 demonstrated a stronger association with elevated CRP levels compared with serum-based proteolytic markers, suggesting that urinary measurements may more sensitively reflect early inflammatory activation. Importantly, CRP was used in this study as a surrogate marker of systemic inflammation rather than as a measure of clinical severity. Consequently, these findings should be interpreted as reflecting differences in early inflammatory response and biological activation patterns, and not as evidence of prognostic superiority for predicting clinical severity or adverse outcomes in acute pancreatitis.

### 4.1. Key Findings and Physiological Explanation

Our findings support and extend the recognized function of trypsinogen activation products as key factors in the pathophysiology of AP. Moderate Bayesian support for a positive relationship between serum TAP and CRP (BF_10_ = 8.42, r = 0.41) corresponds with the idea that systemic inflammation is closely associated with the intraacinar proteolytic cascade [17,18]. Significantly, the mediation analysis provides a statistical pathway: a considerable part (44% for amylase, 42.6% for lipase) of the association between systemic inflammation (CRP) and pancreatic enzyme elevation is accounted for by TAP and trypsin-2. Crucially, this model identifies a temporal and statistical mediation, not a direct mechanistic sequence. From a pathophysiological standpoint, CRP is a downstream acute-phase reactant, not an upstream driver of trypsinogen activation. Therefore, this analysis suggests that the level of systemic inflammation, as captured by CRP, is quantitatively linked to the magnitude of the intrapancreatic proteolytic cascade (reflected by TAP/trypsin-2) and its downstream effects on enzyme secretion [19]. The more pronounced mediation by serum TAP for amylase indicates a more direct link between trypsinogen activation and amylase release, while lipase appears to be affected by both TAP and trypsin-2 through possibly separate pathways [20].

A critical finding is the compartmental divergence of biomarker information. While serum TAP was correlated with inflammation, serum trypsin-2 showed moderate evidence against a correlation with CRP (BF_10_ = 0.45). Urinary trypsin-2 was a consistent and strong predictor of elevated CRP levels in all supervised models, suggesting its value in assessing the magnitude of the early inflammatory response rather than definitive clinical severity. This discrepancy can be interpreted pathophysiologically: serum levels reflect a snapshot of enzyme release and clearance, influenced by rapid kinetics and possible protein binding. Urinary excretion, however, represents an integrated, time-mediated clearance of lower molecular weight fragments, such as TAP, and filtered enzymes, such as trypsin-2 [21,22].

Important Caveat: The interpretation of urinary trypsin-2 as a superior predictor must be heavily qualified. Urinary biomarker levels are a product of their serum concentration, glomerular filtration rate, and tubular function. As we lack data on systemic renal function (serum creatinine/eGFR), we cannot determine to what extent the elevated urinary trypsin-2 levels reflect increased pancreatic production versus decreased renal clearance. Therefore, while its predictive value in our cohort is notable, urinary trypsin-2 should be viewed as a pragmatic but non-specific composite signal of both pancreatic injury and renal handling until studied in cohorts with comprehensive renal function data.

### 4.2. Identification of Novel Patient Phenotypes

The unsupervised K-means clustering based solely on four biomarker measurements revealed three distinct phenotypic clusters with clear clinical correlates. This data-driven subtyping captures heterogeneity often missed by conventional scoring systems.

- Cluster 1 (“Low Biomarker Profile”) represents patients with low biomarker levels in all compartments, younger age, and low inflammation. This profile may correspond to cases with minimal trypsinogen activation.
- Cluster 2 (“Elevated Serum Biomarker Profile”) is characterized by elevated serum biomarkers but only moderate urinary levels. This may indicate significant release of pancreatic enzymes into the circulation without commensurate renal clearance, possibly due to variations in renal function or the timing of sample collection.
- Cluster 3 (“High Serum and Urine Biomarker Profile”) exhibits high levels of biomarkers in both serum and urine, particularly urinary trypsin-2, along with the highest CRP and the oldest age in our cohort. This cluster identifies a distinct phenotype characterized by pronounced biomarker elevation in both compartments. Significant differences in CRP (*p* = 0.032) and age (*p* = 0.047) between clusters suggest these subgroups capture biologically meaningful variation. This exploratory, biomarker-based stratification could serve as a hypothesis-generating complement to traditional scores, meriting further validation against clinical outcomes in larger studies.

This unsupervised stratification reveals distinct pathophysiological states based on early biomarker release and clearance. However, in the absence of clinical outcome data, the association of these subgroups with actual disease severity (e.g., persistent organ failure, mortality) is unknown and represents a critical avenue for future validation.

### 4.3. Predictive Modeling and Clinical Translation

Supervised learning models robustly identified urinary trypsin-2 and age as the two main predictors of elevated CRP levels. It is crucial to distinguish that our models predict a biomarker of inflammation (CRP), not direct clinical severity (e.g., persistent organ failure or mortality). Therefore, while urinary trypsin-2 is a strong indicator of the early inflammatory burden, its utility for forecasting clinical disease progression is not established here and remains a critical objective for future research.

These findings have direct translational implications:

These findings offer insights for future research and potential translational implications:

Biomarker Prioritization for Inflammation Assessment: For early assessment of the inflammatory burden, urinary trypsin-2 should be prioritized as it provides the most informative signal regarding CRP elevation.

Age-Adjusted Interpretation: The strong importance of age requires that any interpretation of TAP and trypsin-2 levels, particularly in the context of inflammation, considers the patient’s age.

Toward a Multimodal Assessment: Our results support the concept of an integrated early assessment combining a key urinary biomarker (trypsin-2) with patient age. Future studies are needed to determine if this panel, when validated against hard clinical endpoints like persistent organ failure, can improve early clinical triage.

### 4.4. Advantages and Disadvantages

The main advantages of this research are its comprehensive analytical strategy, which combines various advanced statistical and machine learning techniques within a clinical cohort, together with direct analysis of serum and urine compartments. The use of Bayesian analysis provides a detailed understanding of the strength of the evidence, while mediation analysis examines causal hypotheses [23,24,25].

Outcome Definition: The primary outcome of our predictive models was CRP, a surrogate marker of inflammation. This is a study limitation, as CRP is not synonymous with clinically defined severe acute pancreatitis (e.g., according to the Revised Atlanta Classification). Our claims are therefore strictly confined to the prediction of inflammatory intensity, not definitive clinical severity. Prospective validation against hard clinical endpoints is essential to translate these findings into a clinical prognostic tool.

The most important limitation of this study is the absence of key clinical data that anchor acute pancreatitis research. Our retrospective dataset did not include standardized information on disease etiology, Revised Atlanta Classification severity, organ failure status, ICU admission, presence of pancreatic necrosis, or interventions performed. Consequently, we cannot correlate the identified biomarker patterns or machine learning predictions with these fundamental clinical outcomes. Our study is therefore a pathophysiological exploration; its clinical utility remains entirely hypothetical and must be evaluated in future prospective cohorts with comprehensive clinical annotation.

Several limitations of the present study should be acknowledged. First, the modeled outcome was C-reactive protein (CRP), which was used as a surrogate marker of early systemic inflammatory response rather than as a validated measure of clinical severity. Consequently, this study does not allow for conclusions regarding the prediction of established severity outcomes such as persistent organ failure, need for intensive care, or mortality. Second, the clustering and regression analyses were exploratory in nature and based on early biomarker measurements, without external validation against clinical endpoints. Therefore, the identified biomarker-defined phenotypes should be interpreted as reflecting biological patterns of early inflammatory activation rather than formal severity stratification.

Causal Inference: The mediation analysis, while insightful, is based on cross-sectional data measured at a single 24 h time point. It establishes a statistical pathway but cannot confirm a direct mechanistic or temporal causal sequence where CRP drives trypsinogen activation. CRP is a robust downstream marker of inflammation, and its association with TAP/trypsin-2 likely reflects their parallel rise in response to the initial pancreatic insult, not a cause-effect relationship. Future studies with frequent serial measurements from symptom onset are needed to delineate true temporal and causal hierarchies.

Timing of Biomarker Measurement: Our study defines ‘early’ biomarkers as those measured within 24 h of hospital admission. This is a significant limitation, as the critical variable for TAP and trypsin-2 kinetics is the time from symptom onset. The lack of standardized symptom-onset-to-sampling time introduces heterogeneity and may obscure the true peak or trajectory of these biomarkers. Consequently, our findings represent a pragmatic ‘real-world’ snapshot early in the hospitalization course, but they cannot inform on the very early in the hospital course (e.g., first 6–12 h) pathophysiological window. Future prospective studies should mandate serial sampling timed from symptom onset to accurately capture the kinetic profiles of these proteolytic markers.

Major Confounding by Unmeasured Renal Function: The most significant limitation affecting the interpretation of our key finding is the complete absence of data on systemic renal function (serum creatinine, estimated glomerular filtration rate, AKI status). Consequently, we could not adjust for renal impairment, a critical determinant of urinary biomarker levels.

Furthermore, a key limitation is that our models predict CRP, a marker of systemic inflammation, and not direct clinical severity endpoints such as persistent organ failure, necrosis, or mortality. Therefore, while urinary trypsin-2 is a robust predictor of inflammatory intensity in our cohort, its utility for predicting clinical severity requires prospective validation against these harder outcomes.

Finally, while we employed robust internal validation techniques, the reporting of our prediction models, though detailed in key aspects, is a summary. A comprehensive TRIPOD checklist—fully detailing all elements of predictor handling, model development, and performance assessment is not provided herein but would be essential for formal clinical implementation and external validation.

## 5. Conclusions

In conclusion, this comprehensive study indicates that initial assessments of TAP and trypsin-2, particularly in urine, provide significant pathophysiological and prognostic insights in acute pancreatitis. We present evidence for their mediating role in the relationship between inflammation and pancreatic injury, discover novel biomarker-based patient subgroups with unique clinical characteristics, and identify urinary trypsin-2 as a promising but confounded early signal associated with inflammatory burden in AP, whose relationship to pancreatic injury versus renal clearance requires urgent clarification in studies with renal function data.

This study demonstrates that early proteolytic biomarker patterns, particularly urinary trypsin-2, are strongly associated with systemic inflammatory burden as reflected by CRP levels in acute pancreatitis. The integration of serum and urinary biomarkers allowed for the identification of distinct biomarker-defined phenotypes, highlighting heterogeneity in early inflammatory activation. These findings support the potential value of urinary proteolytic markers as early indicators of biological response rather than validated predictors of clinical severity. Further studies incorporating established clinical outcomes are warranted to determine the prognostic implications of these biomarker patterns and confirm urinary trypsin-2 as a significant early indicator of the systemic inflammatory response in acute pancreatitis. Future large-scale, prospective studies are warranted to validate whether this biomarker panel, potentially combined with clinical scores, can accurately predict clinical disease severity and progression.

Future studies must validate these proteolytic biomarker panels against hard clinical endpoints in well-characterized cohorts to determine their true utility in precision-based diagnostic and prognostic frameworks.

## Figures and Tables

**Table 1:** Demographic and Biochemical Profile.

Parameter	Mean ± SD	Median (IQR)	Range	Missing Values
**Age (years)**	48.3 ± 16.7	46.0 (34–57)	20–81	0%
**Glucose (mg/dL)**	105.2 ± 47.8	97.5 (78–125)	66–269	25.9%
**CRP (mg/L)**	12.6 ± 13.3	7.5 (2.2–20.9)	0.1–40.7	7.4%
**Amylase (U/L)**	647.4 ± 764.2	368.5 (144–972)	43–2288	37.0%
**Lipase (U/L)**	1768.9 ± 2406.5	690.0 (242–1485)	6–7942	38.9%
**TAP serum (ng/mL)**	4.63 ± 2.50	3.92 (2.87–5.92)	1.21–11.17	0%
**TAP urine (ng/mL)**	6.43 ± 9.40	1.35 (0.08–5.42)	0–42.19	0%
**Trypsin-2 serum (pg/mL)**	2790.7 ± 4785.2	486.2 (148.1–1734.0)	23.9–15,707.3	0%
**Trypsin-2 urine (pg/mL)**	8800.4 ± 10,627.1	5533.8 (143.8–5903.5)	29.1–38,662.7	0%

**Table 2:** Bayesian Correlation Evidence (BF_10_) and Effects.

Biomarker Pair	BF_10_	Evidence Strength	Posterior r Mean	95% Credible Interval	Probability Direction
**TAP serum vs. CRP**	8.42	Moderate	0.41	(0.18, 0.60)	99.2% positive
**TAP serum vs. Amylase**	2.15	Anecdotal	0.28	(0.02, 0.51)	97.8% positive
**TAP serum vs. Lipase**	1.87	Anecdotal	0.26	(−0.01, 0.50)	96.5% positive
**Trypsin-2 serum vs. CRP**	0.45	Moderate against	−0.15	(−0.40, 0.11)	85.3% negative
**Trypsin-2 serum vs. Lipase**	3.21	Moderate	0.34	(0.09, 0.55)	98.9% positive
**Trypsin-2 urine vs. Amylase**	6.89	Moderate	0.39	(0.15, 0.59)	99.1% positive
**Age vs. TAP serum**	12.75	Strong	0.47	(0.25, 0.65)	99.9% positive
**Glucose vs. Trypsin-2 urine**	0.32	Moderate against	−0.22	(−0.46, 0.04)	94.8% negative

**Table 3:** Mediation Analysis Results (Standardized Coefficients).

Pathway	Direct Effect (c’)	Indirect via TAP Serum	Indirect via Trypsin-2 Serum	Total Indirect	Total Effect (c)	Proportion Mediated
**CRP → Amylase**	0.28 *	0.15 **	0.07	0.22 **	0.50 ***	44.0%
**CRP → Lipase**	0.31 **	0.11 *	0.12 *	0.23 **	0.54 ***	42.6%

*** *p* < 0.001, ** *p* < 0.01, * *p* < 0.05; Bootstrapped CI with 5000 resamples.

**Table 4:** Cluster Characteristics and Clinical Profiles.

Characteristic	Cluster 1 (*n* = 19)	Cluster 2 (*n* = 22)	Cluster 3 (*n* = 13)	ANOVA *p*-Value
**TAP serum (ng/mL)**	2.98 ± 1.34	4.28 ± 1.85	8.21 ± 1.94	<0.001
**TAP urine (ng/mL)**	1.62 ± 2.51	4.01 ± 6.32	18.26 ± 13.58	<0.001
**Trypsin-2 serum (pg/mL)**	548.3 ± 498.1	2188.7 ± 2521.4	8967.4 ± 7583.2	<0.001
**Trypsin-2 urine (pg/mL)**	3125.4 ± 3832.1	7479.8 ± 7255.6	20,980.3 ± 15,124.7	<0.001
**CRP (mg/L)**	8.2 ± 8.1	11.9 ± 13.5	20.8 ± 16.4	0.032
**Age (years)**	43.1 ± 15.2	47.8 ± 16.1	57.3 ± 17.8	0.047

**Table 5:** Model Performance Comparison.

Metric	Elastic Net	Random Forest	Baseline (Mean)
**RMSE**	0.82	0.79	1.15
**R^2^**	0.49	0.53	0.00
**MAE**	0.61	0.58	0.89

**Table 6:** Feature Importance Rankings.

Rank	Elastic Net (Standardized β)	Random Forest (Gini Importance)
**1**	Trypsin-2 urine (β = 0.38)	Trypsin-2 urine (0.32)
**2**	Age (β = 0.27)	Age (0.28)
**3**	TAP serum (β = 0.19)	TAP serum (0.18)
**4**	Glucose (β = −0.15)	Glucose (0.14)
**5**	TAP urine (β = 0.08)	Trypsin-2 serum (0.05)
**6**	Trypsin-2 serum (β = 0.00)	TAP urine (0.03)

## Data Availability

The data presented in this study are available on request from the corresponding author due to the privacy of the data.
